# Patients Living Longer With Metastatic Colorectal Cancer

**Published:** 2016-04-01

**Authors:** John L. Marshall, Robin Sommers

**Affiliations:** Georgetown Lombardi Cancer Center in Washington, DC and Dana-Farber Cancer Institute, Boston, Massachusetts

Advances in the treatment of metastatic colorectal cancer have greatly improved survival for these patients, and novel approaches on the horizon are particularly exciting, according to John L. Marshall, MD, of Georgetown Lombardi Cancer Center in Washington, DC, who described the treatment landscape for this malignancy at 2015 JADPRO Live at APSHO.

Colorectal cancer (CRC) is now being recognized as not just one disease, but a cancer with a number of molecular subtypes. It is classified as microsatellite stable (MSS) or unstable (MSI) and as RAS wild-type or mutant; then it is classified anatomically by colon vs. rectum and right side vs. left side of the colon. Most recently, gene profiles and stool flora types are being explored, and researchers are studying how the environment (particularly foods) interacts with the microflora to affect CRC risk.

## SURVIVAL INCREASED IN METASTATIC DISEASE

"Metastatic disease that can be resected can often be cured, not by chemotherapy but by surgery or radiofrequency ablation," Dr. Marshall indicated. Unfortunately, most cases are not that simple. With these patients, he continued, "We have to play an elegant chess game, as we have many new options. Playing these chess pieces wisely, we are actually seeing patients live more than 3 years with metastatic disease."

The multiplicity of options means that many patients move from first-line to fourth-line therapy. By exposing patients to as many effective drugs as possible, survival is increased (Figure).

**Figure:**
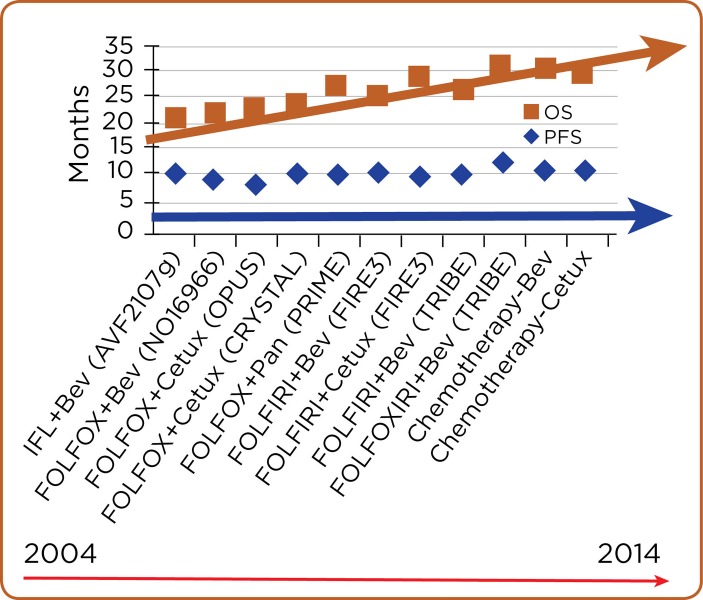
Although overall survival continues to improve, progression-free survival has been mostly stable with first-line therapy in the chemobiologic era. Courtesy of Axel Grothey. Information from Bokemeyer et al. (2011); Douillard et al. (2013); Falcone et al. (2013); Heinemann et al. (2013); Hurwitz et al. (2004); Saltz et al. (2008); Van Cutsem et al. (2011).

The concept of maintenance therapy, now common in the treatment of many different malignancies, was established in CRC by the European CAIRO3 study (Simkens et al., 2015). After completion of chemotherapy, continuous dosing of capecitabine plus bevacizumab (Avastin) led to a doubling in median progression-free survival time, from 4.1 months to 8.5 months (hazard ratio [HR] = 0.44; *p* < .00001), and gave patients a treatment break. "This is quickly becoming a standard treatment," said Dr. Marshall.

Also now established is testing for RAS gene status, which guides treatment selection. *RAS* mutations are observed in 60% of patients with CRC, and these patients typically do not respond to treatment with epidermal growth factor receptor (EGFR) inhibitors.

For patients with nonmutated, i.e., wild-type RAS, disease, the key question has been whether to combine chemotherapy with an anti-EGFR agent or with a drug targeting the vascular endothelial growth factor (VEGF). Two clinical trials reached different conclusions.

The European FIRE-3 trial evaluated first-line treatment of 592 patients with FOLFIRI (irinotecan/fluorouracil [5-FU]/leucovorin) plus either cetuximab or bevacizumab and concluded that anti-EGFR therapy with cetuximab is preferred (Heinemann et al., 2014). Median progression-free survival was about 10 months in each arm, but overall survival was 28.7 months with cetuximab vs. 25.0 months with bevacizumab (HR = 0.77; *p* = .017). Further mutational analysis revealed that 15% of the population had *RAS* mutations not identified originally, and in the subsequent "all–RAS-wildtype" population (which excluded another 15% of potential nonresponders), overall survival increased to 33.1 months with cetuximab.

However, a similar but larger US study of 1,137 patients, CALGB/SWOG 80405, concluded that cetuximab and bevacizumab were equivalent when combined with FOLFIRI or FOLFOX (oxaliplatin/5-FU/leucovorin) in first-line treatment of metastatic RAS wild-type CRC (Venook et al., 2014). Median progression-free survival was approximately 11 months, and overall survival was approximately 32 months in each arm.

"If you use all of the chess pieces, you actually see no difference in one drug versus the other," Dr. Marshall noted. "So in the United States, there still is a bias to use anti-VEGF–type drugs in frontline, even in the *RAS* wild-type patient, whereas in Europe, there tends to be more use of the anti-EGFR drugs."

## NEXT ADVANCE: IMMUNOTHERAPY

"The biggest news for years in CRC was presented at ASCO 2015," Dr. Marshall said, referring to the study of a drug targeting the programmed cell death protein 1 (PD-1).

Explaining how immunotherapy works in CRC, he noted that although 80% of CRC evolves from adenomatous polyps, 20% do not. These tumors have a completely different biology, one that is related to mismatch repair (MMR) deficiency, a mechanism of DNA repair. Patients with MMR-deficient (also known as MSI-high) tumors seem exquisitely sensitive to PD-1 blockade, according to Dr. Marshall.

In the study presented at ASCO 2015, which was also published in *The New England Journal of Medicine* (Le et al., 2015), essentially all patients with MMR-deficient CRC responded to immunotherapy, with many demonstrating prolonged disease control. Median overall and progression-free survival had not been reached at the time of the report. All patients with MMR-proficient tumors failed to respond to immunotherapy and had limited survival.

"We see durable responses, so this is a dramatic impact in the right patients. All patients with CRC are now being tested for MSI status, in hopes of treating them with these agents," revealed Dr. Marshall.

## PUTTING THE PIECES TOGETHER

Dr. Marshall emphasized that not all treatments make patients with CRC better. Outcome tends to be measured in survival time; however, toxicity, quality of life, and cost of care are factors that must be factored into decision-making, he added.

Increasingly, it is becoming important to achieve "value" of care. "This is how we are going to be judged, going forward, as a new metric in the world of cancer," said Dr. Marshall. Rapid discovery of more efficacious and more cost-effective cancer treatments must occur, and underserved populations must gain access to them. Providing global cancer care with value will mandate that the oncology community "come together, listen to each other, respect what we hear, find the common threads, and weave a new fabric," Dr. Marshall concluded.

The field of precision medicine will help accomplish these goals, he predicted. This will include prospective genetic profiling on patients, sharing of this information, and designing drug development and selection based on these profiles.

## IMPORTANCE OF MULTIDISCIPLINARY CARE

Dr. Sommers, DNP, ANP-BC, AOCNP of Dana-Farber Cancer Institute, Boston, Massachusetts described a challenging case that exemplified the need for a multidisciplinary team approach to metastatic colorectal cancer. "Determining the best treatment option in colorectal cancer is very difficult, and multidisciplinary care is the standard now," Dr. Sommers said. Oncologists, advanced practitioners (APs), surgeons, radiation oncologists, genetic counselors, nutritionists, social workers, and other professionals can all lend expertise in developing the treatment plan. This should take into account the patient’s medical, physical, and supportive care needs, she added.

"Treatment is no longer just the responsibility of the solo oncologist, but the team of multiple professionals," declared Dr. Sommers. She emphasized that treatment selection should take into account the patient’s comorbid conditions, performance status, financial concerns, social support, occupation, and personal preferences.

For example, preexisting conditions have an impact on outcomes, she noted. "With all the drugs now available for metastatic CRC, for example, should you consider FOLFOX, which carries the potential for oxaliplatin-induced neuropathy, in a diabetic who may already have some baseline neuropathy? And in a patient with hypertension, should you consider bevacizumab frontline?"
